# Proteomics in Cardiovascular Deaths, a Postmortem Pilot Study: The Diagnostic Efficacy of α-1 Antitrypsin and Apolipoprotein A-IV in Ischemic and Congestive Deaths

**DOI:** 10.3390/diagnostics16081192

**Published:** 2026-04-16

**Authors:** Marina Invernón Monedero, María Esther Pérez Artiago, Juan Pedro Hernández del Rincón, María Dolores Fuentes, María D. Pérez-Cárceles, Eduardo Osuna, Diana Hernández-Romero

**Affiliations:** 1 Department of Legal and Forensic Medicine, Biomedical Research Institute (IMIB), Regional Campus of International Excellence “Campus Mare Nostrum”, Faculty of Medicine, University of Murcia, 30100 Murcia, Spain; 2 Institute of Legal Medicine and Forensic Science of Murcia, 30003 Murcia, Spain

**Keywords:** forensic pathology, sudden cardiac death, ischemic heart disease, proteomics, post-mortem biomarkers, α-1 antitrypsin, apolipoprotein A-IV

## Abstract

**Background/Objectives**: Determining the cardiovascular cause of death, particularly distinguishing ischemic from congestive mechanisms, remains challenging in forensic practice, especially in early ischemia without definitive histological findings. Proteomic techniques and molecular profiling may provide complementary diagnostic information beyond conventional autopsy. **Methods**: We applied an untargeted high-resolution proteomic approach to postmortem cardiac tissue samples from cardiovascular (ischemic and congestive) and non-cardiovascular deaths. Identified proteins were analyzed using bioinformatic and differential expression workflows. Selected candidates were evaluated in peripheral blood samples for translational validation using statistical modeling, including regression analyses and receiver operating characteristic (ROC) curve assessment. **Results**: A total of 572 proteins were identified. Although no proteins fulfilled strict exclusivity criteria for a single cause-of-death group, differential expression analysis revealed distinct molecular patterns distinguishing ischemic, congestive, and non-cardiovascular deaths. Thirty-one proteins were differentially expressed between ischemic and congestive cases, including α-1 antitrypsin (AAT), plasma levels did not demonstrate statistically significant discrimination. In contrast, plasma Apolipoprotein A-IV (ApoA-IV) levels were significantly associated with ischemic death in regression models, and ROC analysis yielded a cutoff point with complete separation between ischemic and selected non-cardiovascular cases. However, the limited sample size warrants cautious interpretation due to potential overfitting. **Conclusions**: Postmortem cardiac proteomic profiling reveals biologically coherent molecular signatures associated with different cardiovascular causes of death. Although further validation in larger independent cohorts is required, ApoA-IV emerges as a promising candidate biomarker for ischemic cardiac death. Multimarker proteomic strategies may complement traditional autopsy to enhance diagnostic accuracy in forensic investigations, particularly in cases with equivocal morphological findings.

## 1. Introduction

Forensic diagnosis of sudden cardiac death (SCD) presents significant challenges and remains a complex investigation. Although autopsy and histopathological analysis are fundamental components of the evaluation, approximately 10–15% of cases show no identifiable cause even after a thorough postmortem assessment and are therefore categorized as “autopsy-negative sudden unexplained deaths” [1].

Proteomics—the large-scale study of proteins—has emerged as a key approach to elucidate the molecular mechanisms underlying cardiovascular diseases, the leading cause of death worldwide. By enabling the simultaneous identification and quantification of thousands of proteins, proteomic technologies provide insights into disease pathways and biomarkers not captured by traditional risk factors or genomic approaches, improving risk stratification and supporting clinical decision-making [2,3,4,5]. By revealing specific protein alterations linked to cardiovascular pathology, proteomics is advancing the discovery of early diagnostic markers and therapeutic targets, thereby offering a promising strategy to reduce cardiovascular mortality.

Proteomic studies have highlighted α-1 antitrypsin (AAT) and apolipoprotein A-IV (ApoA-IV) as proteins of interest in cardiovascular pathology and outcomes. AAT, an acute-phase serine protease inhibitor involved in inflammatory regulation, shows altered expression in certain proteomic analyses and has been examined as a potential biomarker for adverse cardiovascular events; higher plasma AAT levels have been linked to worse clinical outcomes in some cohorts, likely reflecting systemic inflammation and vascular stress relevant to cardiovascular death risk [6,7]. Meanwhile, genetic studies and proteomic profiling suggest that ApoA-IV plays multifaceted roles in lipid metabolism, anti-atherogenic processes, and thrombosis modulation. ApoA-IV concentrations have been inversely associated with coronary heart disease risk independent of conventional lipid measures, indicating a potential protective association with cardiovascular events and mortality when profiled alongside other apolipoproteins in mass spectrometry-based studies [8]. Additionally, targeted proteomic experiments in forensic contexts have identified apolipoprotein family members—including ApoA-IV—and AAT as differentially expressed between diagnostic groups, underscoring the utility of proteomic approaches for identifying proteins that may help distinguish causes of death and associated pathologies [9]. Although direct proteomic evidence linking ApoA-IV and AAT specifically to cardiovascular deaths is still emerging, their involvement in inflammation, lipid transport, and vascular homeostasis makes them strong candidates for further investigation in proteomics-driven cardiovascular research.

We proposed to investigate the utility of MS-based spectrometry to identify biomarkers that add relevant information in the postmortem diagnosis of cardiovascular death. We hypothesized that investigating the proteomic profile in forensic cases may be a valuable tool to improve our knowledge of the pathophysiological differences between ischemic and congestive deaths. The detection of CV biomarkers, associated with ischemic or congestive processes, could provide clinically relevant information, potentially contributing not only to forensic diagnosis but also to the clinical diagnosis, monitoring and management of cardiovascular patients.

## 2. Materials and Methods

### 2.1. Forensic Cases and Sample Collection

For the recruitment of forensic cases, successive cases of sudden cardiac death (SCD) were included at the Institute of Legal Medicine and Forensic Sciences of Murcia. These cases were differentiated after conventional autopsy into the following groups based on the cause of death:

G1. Ischemic cardiovascular death. G2. Congestive cardiovascular death. G3. Non-cardiovascular death with thoracic trauma. G4. Non-cardiovascular death without thoracic trauma.

The cases included in this study had a postmortem interval (PMI) ≤ 24 h and were stored in a refrigerated chamber (≤4 °C) prior to autopsy. We excluded cases with any pre-existing pulmonary pathologies, neoplastic or chronic inflammatory diseases, sepsis or poisoning. Additional exclusion criteria included PMI > 24 h or storage temperatures above 4 °C. The study was approved by the Research Ethics Committee of the University of Murcia (approval ID: 2323/2019; approval date: 14 March 2019).

Cases were classified based on comprehensive evaluation, including clinical history, circumstances of death, and autopsy findings. Within cardiac deaths, cases were categorized as ischemic when histological evidence of myocardial necrosis and significant coronary artery stenosis were present, or as congestive when morphological and histological features consistent with chronic cardiac dysfunction were identified (e.g., ventricular dilatation, cardiomegaly, or myocardial degeneration). Control cases included non-cardiac deaths without macroscopic or microscopic evidence of myocardial ischemia. All analyses were conducted according to SCD guidelines by pathologists blinded to clinical data and cause of death. Autopsies were performed in accordance with the Council of Europe Recommendation No. (99)3 for the harmonization of medico-legal autopsy procedures. Relevant clinical history related to cardiovascular disease was recorded when available, including prior embolic or hemorrhagic events, pharmacological treatment, previous stroke, myocardial infarction, and other pertinent conditions.

Biological samples were collected according to a standardized protocol. Blood was drawn into Vacutainer^®^ tubes for serum, EDTA plasma, and citrated plasma. Hemolyzed samples were excluded. Plasma and serum were obtained by centrifugation at 3500× *g* for 15 min and stored at −80 °C until analysis.

Tissue samples were collected during autopsy following a standardized approach. When present, areas with macroscopic features suggestive of ischemia (e.g., necrosis or hemorrhage) were preferentially sampled. In the absence of visible lesions, systematic sampling was performed from predefined anatomical regions, specifically the upper third of the interventricular septum and the upper third of the left ventricular free wall. The same regions were sampled in all cases, including controls, to ensure comparability and minimize bias. Tissue samples were preserved either in RNAlater solution and stored at −80 °C or fixed in formaldehyde and kept at room temperature until analysis.

### 2.2. Proteomic Technique Procedure

Heart tissue samples were thawed on ice, weighed, and washed with phosphate-buffered saline (PBS). For tissue protein extraction, ~100 mg of heart tissue was minced into 2 mm pieces. The samples were then homogenized using a FastPrep-24 5G homogenizer (MP Biomedicals, Irvine, CA, USA) with a validated protocol for heart samples as follows: 40 s cycles were carried out at a speed of 6.0 m/s. The four cycles were performed with 5 min intervals between each, and the samples were placed on ice for preservation. The samples were then centrifuged for 15 min at 17,000× *g* at 10 °C, yielding a protein-rich supernatant. Protein quantification was performed using the Coomassie Plus Bradford Assay Kit (kit ref 23236). Samples were digested with the following standard procedure. Samples were dissolved in 200 µL of 50 mM ammonium bicarbonate buffer pH 8.5 with 0.01% ProteaseMax (Promega Corporation, Madison, WI, USA)). This surfactant enhances the trypsin digestion. Protein samples were reduced by adding 10 mM DTT at 56 °C for 20 min. Then, samples were alkylated by adding 25 mM IAA during a 30 min period at room temperature in the dark. Finally, digestion was performed by adding 1 µg of Trypsin Gold Proteomics Grade (Promega) (octupole 1:100 *w*/*w*) followed by incubation for 16 h at 37 °C. The reaction was stopped with 0.1% formic acid and the supernatants were recovered using a magnetic stand and finally dried using an Eppendorf Vacuum Concentrator model 5301 (Eppendorf AG, Hamburg, Germany).

### 2.3. HPLC-MS/MS Analysis

The separation and analysis of the tryptic digests of the samples were performed with a HPLC/MS system consisting of an Agilent 1290 Infinity II Series HPLC (Agilent Technologies, Santa Clara, CA, USA) equipped with an Automated Multisampler module and a High Speed BinaryPump and connected to an Agilent 6550 Q-TOF Mass Spectrometer (Agilent Technologies, Santa Clara, CA, USA) using an Agilent Jet Stream Dual electrospray (AJS-Dual ESI) interface. Experimental parameters for HPLC and Q-TOF were set in MassHunter Workstation Data Acquisition Software, version M13.1 (Agilent Technologies, Santa Clara, CA, USA).

Dry samples from trypsin digestion were resuspended in 20 µL of buffer A, consisting of water/acetonitrile/formic acid (94.9:5:0.1). The sample was injected onto an Agilent Advance Bio Peptide Mapping HPLC column (2.7 µm, 100 × 2.1 mm, Agilent Technologies) and thermostated at 50 °C, at a flow rate of 0.4 mL/min. This column is suitable for peptide separation and analysis. After the injection, the column was washed with buffer A for 3 min and the digested peptides were eluted using a linear gradient 0–40% B (buffer B: water/acetonitrile/formic acid, 10:89.9:0.1) for 40 min followed by a linear gradient from 40 to 95% B for 8 min. A total of 95% B was maintained for 3 min. Finally, the column was equilibrated under the initial conditions for 6 min before every injection.

The mass spectrometer was operated in positive mode. The nebulizer gas pressure was set to 35 psi, whereas the drying gas flow was set to 14 L/min at a temperature of 300 °C, and the sheath gas flow was set to 11 L/min at a temperature of 250 °C. The capillary spray, nozzle, fragmentor and octupole RF Vpp voltages were 3500 V, 100 V, 360 V and 750 V respectively. Profile data were acquired for both MS and MS/MS scans in extended dynamic range mode at 4 GHz. The MS and MS/MS mass range was 50–1700 *m*/*z* and scan rates were 8 spectra/sec for MS and 3 spectra/sec for MS/MS. Auto MS/MS mode was used with precursor selection by abundance and a maximum of 20 precursors selected per cycle. A ramped collision energy was used with a slope of 3.68 and an offset of −4.28. The same ion was rejected after two consecutive spectra. Data processing and analysis was performed Spectrum Mill MS Proteomics Workbench (Rev B.06.00.201, Agilent Technologies, Santa Clara, CA, USA). Briefly, raw data were extracted under default conditions as follows: unmodified or carbamidomethylated cysteines; [MH]+50–10,000 *m*/*z*; maximum precursor charge of +5; minimum signal-to-noise MS (S/N) 25; detection of 12C signals. The MS/MS search against the appropriate and updated protein database was performed with the following criteria: variable modifications search mode (carbamidomethylated cysteines, STY phosphorylation, oxidized methionine, and N-terminal glutamine conversion to pyroglutamic acid); tryptic digestion with a maximum of 5 missed cleavages; ESI-Q-TOF instrument; minimum matched peak intensity of 50%; maximum ambiguous precursor charge of +5; monoisotopic masses; peptide precursor mass tolerance of 20 ppm; product ion mass tolerance of 50 ppm; and calculation of reversed database scores. Validation of peptide and protein data was performed using auto thresholds. For this purpose, the most up-to-date version of the Uniprot human protein list was used (https://www.uniprot.org/uniprotkb?query=organism_id%3A9606; accessed on 5 September 2025). All the proteomic procedures were performed at the Metabolomics Proteomics Section of the Molecular Biology Service at the University of Murcia.

### 2.4. Validation Experiment

For the validation experiment, we studied plasma samples to evaluate whether differentially expressed proteins can serve as biomarkers for the determination of the cause of death. Plasma samples were obtained as previously described and analyzed at the Proteomics Platform of the Biomedical Research Institute (IMIB) to quantify AAT and ApoA-IV levels by Enzyme-Linked Immunosorbent Assay (ELISA). The principle of this method is based on the formation of immune complexes between the target proteins and specific antibodies, followed by an enzyme-mediated reaction that produces a measurable signal proportional to the concentration of the analyte in the sample. The “N Antisera to Human ApoA-IV” assay (Siemens Healthcare Diagnostics Products GmbH, Marburg, Germany) was used to determine ApoA-IV levels, with intra- and inter-assay coefficients of variation of 2.7% and 2.3%, respectively. For AAT determination, the “N Antisera to Human α-1 Antitrypsin” assay (Siemens Healthcare Diagnostics Products GmbH, Marburg, Germany) was used, with intra- and inter-assay coefficients of variation of 2.7% and 1.9%, respectively.

### 2.5. Statistical Analysis

Each categorical variable was expressed as a frequency (percentage) of cases. Continuous variables were tested for normal distribution by the Kolmogorov–Smirnov test. The normal distributed continuous variables were shown as mean ± SD, and those non-parametrically distributed are shown as median (interquartile range). Differences between groups were assessed by the unpaired Student *t*-test or the Mann–Whitney U test for continuous variables and the ANOVA or Kruskal–Wallis test (as appropriate). Multivariate analysis by linear regression, adjusted by confounding factors such as age and gender, was used to identify the factors independently associated with biomarkers.

Protein differential expression identified by the Uniprot human protein list (https://www.uniprot.org/uniprotkb?query=organism_id%3A9606; accessed on 5 September 2025) was performed with the R package MsnSet.utils (https://github.com/PNNL-Comp-Mass-Spec/MSnSet.utils/tree/master; accessed on 5 September 2025), specifically with a function adapted from the R package limma [10]. To quantify protein abundance, an R script was used to process the data generated in Excel files. Normalization and filtering methods were applied to ensure the accuracy of the analysis. A logarithmic transformation (base 2) was applied to normalize the intensity distribution. Proteins not present in at least 50% of the replicates per condition were excluded. Missing values were imputed using the Missing Not at Random (MNAR) method, adjusted to the nature of the data. The *p*-values were adjusted by the Benjamini–Hochberg (BH) method. Principal component analysis (PCA) was performed with the FactoMineR [11] and factoextra [12] R packages with data scaled to unit variance.

For the concentrations of ApoA-IV, a receiver operating characteristic curve (ROC) was drawn, and the area under the curve was measured using a non-parametric test. The diagnostic performance of a test or the ability of a test to discriminate between causes of death was evaluated using ROC curve analysis. The analysis of ROC curves provides an excellent view. The cut-off point was taken as the point of the ROC curve with the highest sensitivity and lowest 1-specificity (best Youden Index).

## 3. Results

### 3.1. Proteomic Approach

We performed a pilot proteomic experiment with tissue samples from 17 forensic cases (71% men), grouped by diagnosis of death as follows: four ischemic cardiovascular deaths, five congestive cardiovascular deaths, four non-cardiovascular and non-traumatic deaths and four non-cardiovascular thoracic trauma deaths. A description of the most relevant corpse characteristics is shown in Table 1. No statistical differences were obtained for any of the postmortem or clinical variables according to the classified cause of death.

**Figure 1:**
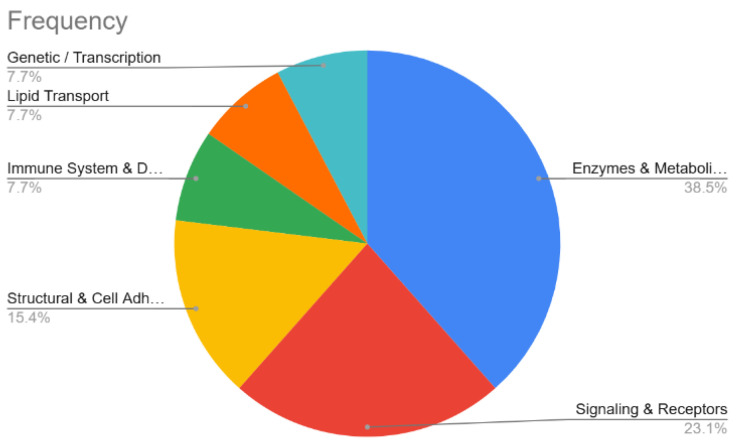
Frequency of proteins exclusively detected in cardiovascular (ischemic and congestive) deaths compared with non-cardiovascular cases, identified by comparative proteomic analysis, classified by their biological function.

A total of 572 proteins were identified from tissue samples (Supplementary Table S1). Analysis performed by cause of death revealed that 22 proteins were identified only in ischemic cases and not in other causes whereas 19 were identified only in congestive samples and not in other causes. However, when the threshold was considered as detection in minimum three of four samples or four of five samples, depending on the total samples in the group, no unique proteins were detected for any isolated cause of death. Hence, we proposed to evaluate unique proteins expressed for CV cases not detected in non-CV deaths in order to find suitable biomarkers for cardiovascular death. A total of 13 proteins, including a protein involved in lipid transport, ApoA-IV, seemed to be only expressed in CV (ischemic and congestive) versus non-CV cases (Table 2, Figure 1).

**Figure 2:**
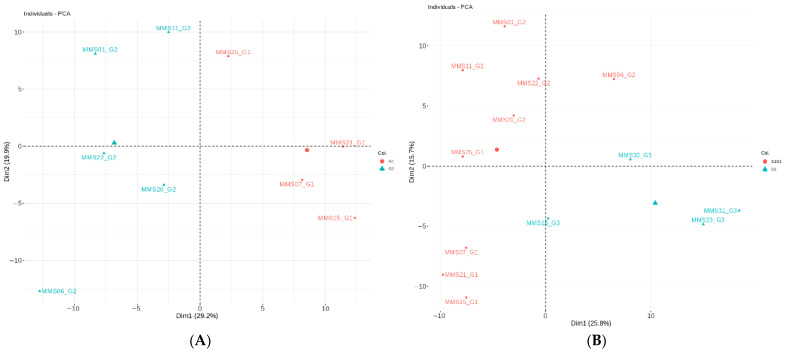

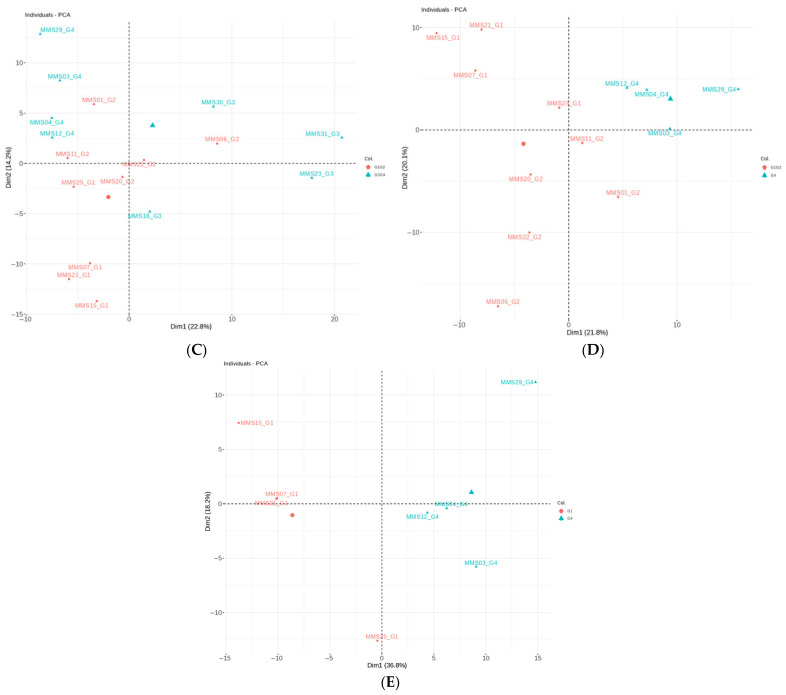
PCA analysis for comparison between groups. (**A**) ischemic vs. congestive; (**B**) CV vs. non-CV non-thoracic trauma; (**C**) CV vs. non-CV samples; (**D**) CV vs. non-CV with thoracic trauma and (**E**) ischemic vs. non-CV with thoracic trauma.

Since protein presence/absence analyses did not reveal proteomic differences among the four groups of deaths, comparisons for differential protein level expression, depending on the cause of death, were performed as indicated in the Methods Section. All the identified proteins were studied according to their annotated biological function, pattern and published pathophysiological pathways. Principal Component Analysis (PCA) was performed for each comparison below, and all tissue samples were correctly grouped according to the cause of death (Figure 2).

When all differentially expressed proteins were analyzed depending on the cause of death, we found 31 proteins that were significantly differently expressed in ischemic cases versus congestive cases. A total of 35 proteins were differentially expressed between ischemic deaths and non-CV thoracic trauma cases, 43 proteins were differentially expressed between CV deaths (both ischemic and congestive) and non-CV non-traumatic deaths, 31 proteins were differentially expressed between CV and non-CV non-thoracic traumatic death and six proteins were differentially expressed between CV and non-CV deaths (Supplementary Table S2).

**Figure 3:**
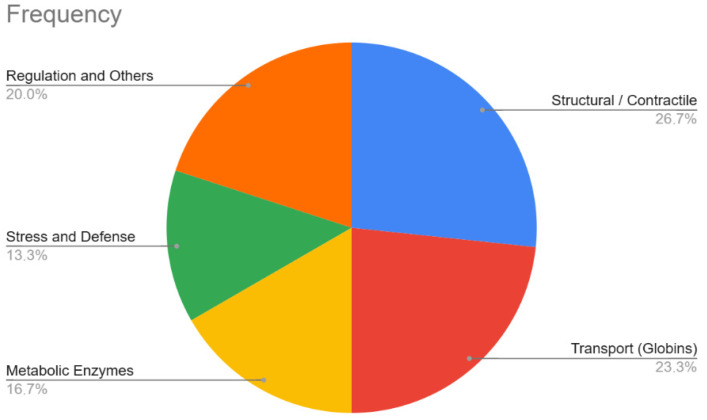
Frequency of proteins differentially between ischemic and congestive cardiac deaths, identified by comparative proteomic analysis classified by their biological function.

As our main objective, we focused on the comparison of differential expressions in proteins between ischemic and congestive cases; we found a significant concentration of blood components (globins) and stress-response proteins here, which could indicate a vascular or blood tissue sample under certain physiological stress conditions. Among these stress-response proteins we found that AAT were differently expressed in ischemic cases when compared with congestive ones (Table 3, Figure 3).

### 3.2. Validation Experiment

**Figure 4:**
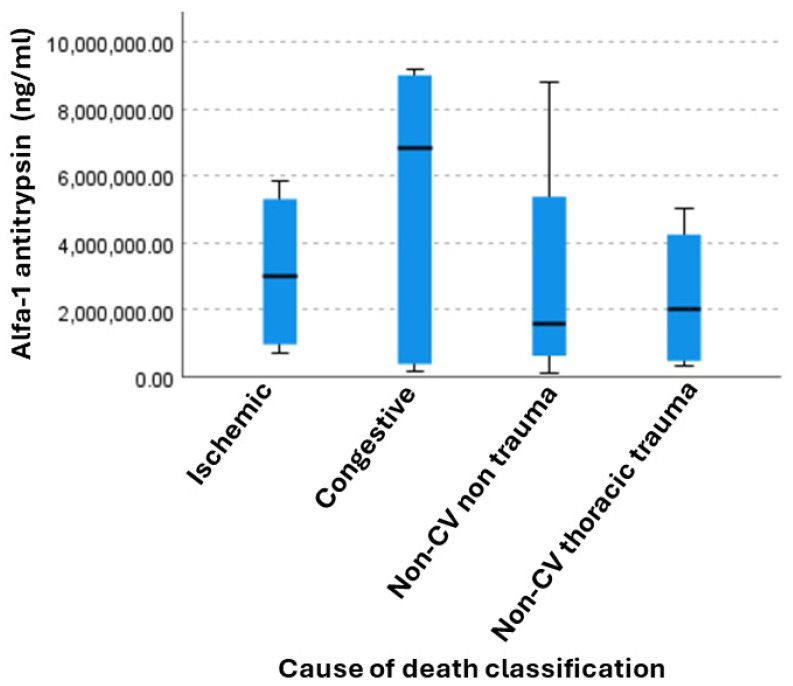
Kruskal–Wallis test conducted to compare the distribution of Alfa-1 antitrypsin levels across independent groups based on the cause of death (*p* = 0.789).

To evaluate suitable usage of AAT as a biomarker of CV deaths, we evaluated the distribution of plasma AAT levels across the four distinct causes of death using a Kruskal–Wallis test for independent samples (Figure 4). Visual inspection of the box plot distribution suggests that the congestive group presented the highest median concentrations, whereas the non-CV non-thoracic trauma group showed the lowest median values. Additionally, both the congestive and the ischemic categories exhibited higher levels compared with non-CV deaths, indicating that Alfa-1 antitrypsin may be a suitable candidate for CV deaths. Despite these visual trends, the post hoc pairwise comparisons revealed no statistically significant differences between any of the diagnostic groups, even after applying the Bonferroni correction to account for multiple testing. Consequently, AAT levels do not differ significantly based on the cause of death classification within this study population (*p* = 0.789).

**Figure 5:**
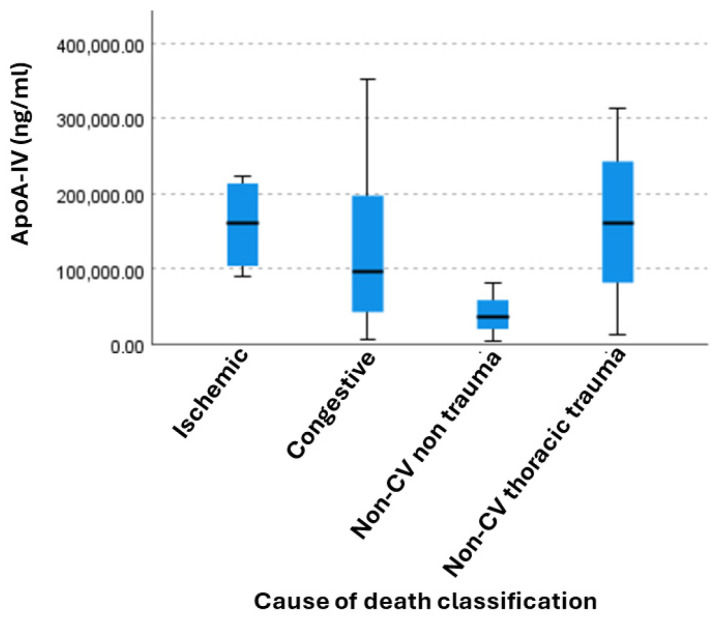
Kruskal–Wallis test conducted to compare the distribution of ApoA-IV levels across independent groups based on the cause of death (*p* = 0.234).

In parallel and taking into account our results in tissue ApoA-IV expression, a Kruskal–Wallis test was conducted to compare the distribution of plasma ApoA-IV across the four independent groups based on the cause of death in order to validate our previous proteomic pilot experiment showing expression in CV cases (Figure 5). We observed a noticeable difference in the medians (represented by the black horizontal lines) across the categories. Non-CV non-thoracic trauma cases showed the lowest median levels of ApoA-IV and the smallest interquartile range, suggesting more uniform, lower values in this category. On the contrary the congestive CV group exhibited the highest variability and higher maximum values compared to the other groups. In addition, ischemic CV and non-CV thoracic trauma groups showed relatively similar median values, indicating similar behavior for the liberation pathways of ApoA-IV into blood in ischemic and thoracic trauma cases. However, we found no statistically significant differences among ApoA-IV levels in the classification groups (*p* = 0.234). Moreover, none of the pairwise comparisons reached statistical significance after applying the Bonferroni correction (adjusted *p* > 0.05 for all comparisons), but a strong trend showing borderline significant difference was observed for ischemic CV vs. non-CV non trauma groups (*p* = 0.051, Table 4).

In light of the aforementioned findings, we sought to further examine the comparison between the ischemic and non-CV non-thoracic trauma cohorts for the biomarker ApoA-IV. A linear regression model was used to evaluate the relationship between the cause of death classification and ApoA-IV levels, confirming a significant association between the cause of death classification and protein levels (*p* = 0.039), specifically highlighting significantly higher concentrations in the ischemic CV group compared to the non-CV non-thoracic trauma group (Table 5).

**Figure 6:**
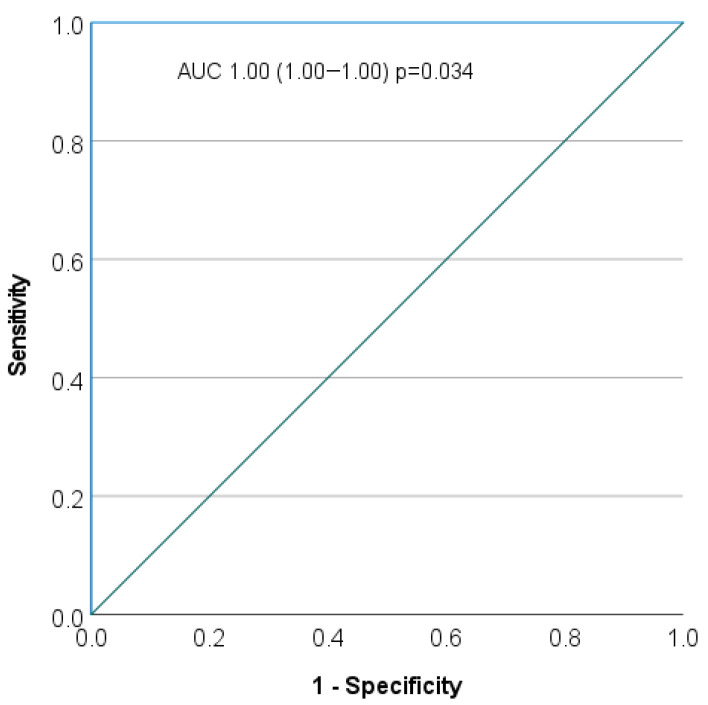
Receiver operating characteristic (ROC) for evaluation of ApoA-IV levels related to the cause of death.

Receiver operating characteristic (ROC) curve analysis was conducted to assess the discriminatory performance of ApoA-IV on serum levels of the included cases according to the cause of death (Figure 6). ApoA-IV demonstrated an area under the curve (AUC) of 1.00 (standard error = 0.00; 95% confidence interval [CI], 1.00–1.00, *p* = 0.034), indicating complete separation between cases (ischemic deaths) and controls (non-CV non-thoracic trauma deaths) within the study sample (Figure 6). The optimal cut-off value, determined by maximizing the Youden Index, was 85,267.86 ng/mL, corresponding to a sensitivity of 100% and a specificity of 100% (Youden Index = 1.00). Although these findings suggest excellent diagnostic performance, the observed perfect discrimination should be interpreted with caution, as such results may reflect sample size limitations or potential overfitting. External validation in independent cohorts is warranted to confirm the robustness and generalizability of these findings.

Logistic regression analysis further confirmed the strong association between high ApoA-IV levels and ischemic death. Likelihood ratio testing demonstrated that the removal of ApoA-IV from the model significantly worsened the model fit (change in −2 log-likelihood = 9.561; df = 1; *p* < 0.002), indicating that the variable provides significant explanatory value beyond the reduced model. Due to the small sample size and the potential presence of quasi-separation, a Firth penalized logistic regression model was applied. ApoA-IV above the cutoff value showed a positive association with the outcome (OR ≈ 5.0), although this did not reach statistical significance (*p* ≈ 0.20) (Table 6). Confidence intervals were wide, reflecting the limited sample size and uncertainty in the estimates. In addition, the model performance was evaluated using leave-one-out cross-validation. The model showed modest discriminative ability, with an AUC of approximately 0.69, an accuracy of 0.63, a sensitivity of 0.75, and a specificity of 0.50.

## 4. Discussion

In this pilot study, we applied an untargeted proteomic approach to postmortem cardiac tissue with the aim of identifying candidate biomarkers that may support the forensic diagnosis of cardiovascular (CV) causes of death. Using high-resolution HPLC-MS/MS analysis and bioinformatic processing based on the UniProt human protein database, we identified a total of 572 proteins across the analyzed samples. Although no proteins fulfilled strict criteria for exclusivity within a single cause-of-death group, differential expression analyses revealed biologically consistent patterns that distinguished ischemic, congestive, and non-cardiovascular deaths. These findings support the hypothesis that molecular profiling can provide complementary information to conventional autopsy in sudden cardiac death (SCD).

### 4.1. Proteomic Profiling and Biological Plausibility

The absence of strictly unique proteins for individual causes of death—when applying more stringent detection thresholds—highlights the biological overlap inherent to terminal cardiovascular pathways. Both ischemic and congestive deaths share final common mechanisms such as hypoxia, metabolic stress, inflammation, and structural myocardial remodeling. Nevertheless, comparative quantitative analyses identified distinct expression signatures. Acute myocardial ischemia has been characterized by rapid metabolic shifts, calcium dysregulation, and activation of proteolytic and inflammatory cascades, even before overt histological necrosis becomes evident [13,14]. In contrast, congestive heart failure typically reflects chronic hemodynamic overload, neurohormonal activation, extracellular matrix remodeling, and sustained inflammatory signaling [15].

In our comparison between ischemic and congestive deaths, 31 proteins were differentially expressed. A notable enrichment of globin chains and stress-related proteins was observed, suggesting differing degrees of hypoxic burden and oxidative stress. Increased detection of hemoglobin subunits and transferrin in ischemic samples may reflect acute vascular leakage and reperfusion-related tissue injury, processes well described in ischemia–reperfusion models [16].

Among stress-response-related proteins, AAT was differentially expressed in ischemic cases compared with congestive cases. AAT is a serine protease inhibitor and acute-phase reactant involved in the modulation of inflammation and tissue protection. Abnormal circulating levels have been associated with cardiovascular disease, the progression of atherosclerosis, and adverse outcomes [17]. Experimental evidence also suggests that AAT exerts tissue-protective and anti-inflammatory effects in ischemic injury models [17,18]. Therefore, its differential myocardial expression in our ischemic cases is biologically plausible. However, its lack of statistically significant discrimination in plasma samples indicates that it may reflect a nonspecific acute-phase response rather than a cause-specific marker.

Additionally, the identification of apolipoproteins, particularly ApoA-IV, in CV but not non-CV tissue samples in the pilot phase is biologically plausible. ApoA-IV is synthesized primarily in the intestine and plays a key role in lipid transport, reverse cholesterol transport, antioxidative activity, and modulation of inflammation [19,20]. Their myocardial detection may reflect altered lipid metabolism, endothelial dysfunction associated with ischemic injury and chronic inflammatory activation accompanying cardiovascular dysfunction. However, further analyses as functional assays or targeted mechanistic studies will corroborate the role of ApoA-IV, since mechanistic insights remain to be fully elucidated.

### 4.2. Plasma Validation and Translational Implications

The validation phase sought to determine whether tissue-level findings could translate into measurable circulating biomarkers. Although plasma α-1 antitrypsin levels showed higher median concentrations in CV deaths, no statistically significant differences were observed after correction for multiple comparisons. Postmortem redistribution, hemolysis, agonal events, and variable inflammatory states may contribute to systemic variability and attenuate discriminatory capacity [21]. Therefore, tissue-level differences do not necessarily correspond to clear plasma discrimination. This phenomenon has been reported in forensic biomarker research, where promising tissue-level findings are not always mirrored in peripheral blood samples [22].

In contrast, ApoA-IV demonstrated potentially relevant discriminatory capacity. Although the global Kruskal–Wallis test did not reveal statistically significant differences across the four groups, a borderline difference emerged in the Bonferroni-corrected pairwise comparison between ischemic CV deaths and non-CV, non-thoracic trauma cases. This apparent discrepancy is likely attributable to methodological differences, as the conservative nature of the Bonferroni correction may limit sensitivity to detect true effects. Importantly, linear regression analysis confirmed a statistically significant association between ischemic death and increased ApoA-IV levels. While these results suggest excellent discriminatory performance, the perfect separation observed must be interpreted cautiously. The limited sample size increases the risk of overfitting and optimistic performance estimates. Overfitting is a recognized concern in biomarker discovery studies, particularly in pilot cohorts without independent validation [23]. In consequence, we applied Firth penalized logistic regression to account for small sample size and quasi-separation, and the observed association was attenuated and no longer statistically significant, with wide confidence intervals indicating substantial uncertainty. Cross-validation showed modest discrimination (AUC ≈ 0.69), suggesting limited predictive value as a standalone marker. These findings should be interpreted cautiously and require validation in larger cohorts. In forensic biomarker research, replication in independent cohorts is essential before clinical or medico-legal implementation. Nonetheless, the concordance between tissue-level proteomic findings and plasma-based statistical modeling strengthens the biological credibility of ApoA-IV as a candidate biomarker for ischemic cardiac death.

### 4.3. Forensic Relevance

The differentiation between ischemic and congestive cardiovascular deaths can be challenging in routine forensic practice, especially in early ischemia without established myocardial necrosis. Conventional autopsy and histology may fail to detect early biochemical alterations occurring within minutes to hours after coronary occlusion [13]. Molecular biomarkers reflecting acute ischemic stress, inflammatory activation, or metabolic shifts could therefore provide valuable complementary evidence [24].

Proteomic approaches have increasingly demonstrated their potential in forensic pathology, particularly for sudden cardiac death investigation [25,26]. Our findings, based on proteomic profiling of cardiac tissue, revealed the expression of different proteins between CV and non-CV deaths and, more specifically, between ischemic and congestive mechanisms. ApoA-IV may reflect acute ischemic metabolic disturbances rather than chronic hemodynamic overload and can be measured in plasma. If validated, such biomarkers could support cause-of-death certification in complex forensic cases, especially when morphological findings are equivocal.

### 4.4. Limitations

Several limitations must be acknowledged. First, the pilot nature and small sample size reduce statistical power and may inflate apparent effect sizes. More restrictive statistical analyses have been applied to obtain a more real-world interpretation of our results. Second, postmortem biological variability—including agonal state, resuscitation attempts, and individual comorbidities—may influence protein expression profiles. Third, although cases were restricted to a postmortem interval under 24 h and preserved under standardized conditions, postmortem proteolysis and redistribution cannot be completely excluded. The absence of external validation limits generalizability. Finally, biological interpretation remains speculative and the mechanistic explanation for the ApoA-IV role needs further functional experiments that are beyond the scope of the present pilot experiment.

### 4.5. Future Directions

Future studies should include larger, multicenter cohorts with independent validation sets to confirm the diagnostic accuracy of ApoA-IV and other candidates. Integration with transcriptomic or metabolomic approaches may further refine mechanistic understanding. In this context, artificial intelligence (AI) may further enhance proteomic analyses by enabling the identification of complex patterns within large-scale datasets, thereby improving diagnostic accuracy and risk stratification in cardiovascular diseases [27]. Moreover, evaluating biomarker performance in challenging subgroups—such as early myocardial infarction without macroscopic lesions—would clarify real-world forensic translational value.

## 5. Conclusions

This study demonstrates that high-resolution proteomic analysis of postmortem cardiac tissue reveals distinct molecular patterns associated with cardiovascular causes of death. Although AAT showed differential tissue expression without significant plasma discrimination, ApoA-IV emerged as a promising candidate biomarker for ischemic cardiac death. Despite the need for external validation, these findings highlight the potential of multimarker proteomic strategies to complement conventional autopsy and improve diagnostic accuracy in forensic pathology.

## Figures and Tables

**Table 1:** Overview of analyzed forensic cases and associated postmortem and clinical variables.

Forensic Case	PMI (h)	Storage Temperature (°C)	Resuscitation	Classified Cause of Death *	Gender ^φ^	Age (Years)
1	3.0	4	NO	2	1	68
2	1.5	4	NO	4	2	48
3	1.0	4	NO	4	1	34
4	2.0	4	NO	2	1	71
5	14.5	4	NO	1	1	42
6	23.0	4	NO	2	2	76
7	2.5	4	YES	4	1	87
8	9.5	4	YES	1	1	44
9	1.5	4	YES	3	2	41
10	9.0	4	YES	2	1	61
11	6.0	4	YES	1	1	55
12	2.5	4	NO	2	1	65
13	4.0	4	NO	3	2	60
14	10.0	4	YES	1	1	61
15	3.5	4	YES	4	1	54
16	3.0	4	YES	3	1	36
17	17.0	4	NO	3	2	65

* Classified cause of death: 1 ischemic, 2 congestive, 3 non-CV non-thoracic trauma, 4 non-CV thoracic trauma. ^φ^ Gender: 1 male, 2 female.

**Table 2:** Functional classification of proteins exclusively detected in cardiovascular (ischemic and congestive) deaths compared with non-cardiovascular cases.

Functional Category	Included Proteins
Enzymes and Metabolism	Alpha-1,3-glucosyltransferase, Palmitoyltransferase, Phosphoglucomutase-1, Sulfurtransferase, Alpha-2-HS-glycoprotein.
Signaling and Receptors	Janus kinase and microtubule-interacting protein 1, Progestin and adipoQ receptor family member 4, Tumor necrosis factor receptor superfamily member 19-like.
Structural and Cell Adhesion	Laminin subunit alpha-2, PDZ and LIM domain protein 3.
Immune System and Defense	Squamous cell carcinoma antigen recognized by T-cells 3.
Lipid Transport	Apolipoprotein A-IV.
Genetic/Transcription	STON1-GTF2A1L readthrough transcript.

**Table 3:** Functional classification of differentially expressed proteins between ischemic and congestive cardiac deaths, identified by comparative proteomic analysis.

Functional Category	Included Proteins
Structural/Contractile	Tropomyosin 1 (Alpha) (CRA_o and isoform 1), Beta-actin-like protein 2, Actin (alpha skeletal, alpha cardiac1, and actin-like), Tropomyosin alpha-3, Laminin subunit beta-2.
Transport (Globins)	Hemoglobin alpha 1 (and fragment), Beta-globin (and fragment), Alpha globin, Hemoglobin subunit beta, G-gamma-hemoglobin, Serotransferrin.
Metabolic Enzymes	Malate dehydrogenase (mitochondrial and cytoplasmic), Insulin-degrading enzyme, ADAMTS 20, Phosphopyruvate hydratase.
Stress and Defense	Heat shock protein beta-1, Heat shock protein beta-6, Alpha-1-antitrypsin, Alpha-1-acid glycoprotein 1.
Regulation and Others	Histone H2AX, ERC protein 2, Coiled-coil domain-containing protein 40, Full-length cDNA clone, LOC100049716, Mutant hemoglobin gene fragment.

**Table 4:** Each row tests the null hypothesis that the distributions of Sample 1 and Sample 2 are equal. Asymptotic significances (2-sided tests) are displayed. The significance level is 0.050.

Sample 1–Sample 2	Test Statistic	Standard Error	Standardized Test Statistic	Sig.
No CVD no thoracic trauma–Congestive	4.933	3.477	1.419	0.156
No CVD no thoracic trauma–No CVD with thoracic trauma	−6.083	3.636	−1.673	0.094
No CVD no thoracic trauma–Ischemic CVD	7.083	3.636	1.948	0.051
Congestive–No CVD with thoracic trauma	−1.150	3.194	−0.360	0.719
Congestive CVD–Ischemic	2.150	3.194	0.673	0.501
No CVD with thoracic trauma–Ischemic	1.000	3.367	0.297	0.766

**Table 5:** Linear regression analysis for ApoA-IV levels.

Coeficients ^a^								
Model		Unstandardized Coefficients		Standardized Coefficients	t	Sig.	95.0% Confidence Interval for B	
		B	Std. Error	Beta			Lower Bound	Upper Bound
	(Constant)	217,819.9	44,848.3		4.857	0.005	102,533.6	333,106.3
	Cause of death	−59,114.5	21,311.5	−0.779	−2.774	0.039	−113,897.6	−4331.6

^a^ Dependent Variable: ApoA-IV.

**Table 6:** Firth penalized logistic regression for the prediction of ischemic death.

Variable				
		β (Coefficient)	Odds Ratio (95% CI)	*p*-Value
	ApoA-IV positive	1.61	5.00 (0.40–78.0)	0.2
Based on conditional parameter estimates				

## Data Availability

The raw data supporting the conclusions of this article will be made available by the authors upon request.

## Supplementary Materials

The following supporting information can be downloaded at https://www.mdpi.com/article/10.3390/diagnostics16081192/s1: Supplementary Table S1. Proteins identified and quantified in the proteomic analysis; Supplementary Table S2. Comparative analysis of differentially expressed proteins among causes of death.

## Abbreviations

The following abbreviations are used in this manuscript:

SCD	sudden cardiac deaths
AAT	α-1 antitrypsin
ApoA-IV	apolipoprotein A-IV
ELISA	Enzime Immunoassay
PCA	Principal Component Analysis
ROC	Receiver operating characteristic
AUC	Area under the curve
